# Mobile computed tomography for the efficient allocation of medical resources in patients with COVID-19 pneumonia: A case report

**DOI:** 10.1097/MD.0000000000027872

**Published:** 2021-11-19

**Authors:** Jae Wan Jung, Hak Ryul Kim, Kwon Ha Yoon, Chul Park

**Affiliations:** a Division of Pulmonology, Department of Internal Medicine, Wonkwang University Hospital, Muwang-ro 895, Iksan-si, Jeollabuk-do, South Korea; b Department of Radiology, Wonkwang University Hospital, Muwang-ro 895, Iksan-si, Jeollabuk-do, South Korea.

**Keywords:** allocation of resources, case report, cone-beam computed tomography, coronavirus 2019, mobile application

## Abstract

**Rationale:**

The highly contagious Coronavirus 2019 (COVID-19) infection raise social and economic burden. Medical staff and resources are being diverted for the care of patients with COVID-19. There are problems for healthcare systems, including burnout syndrome for the medical staff and exhaustion of medical resources.

**Patient concerns:**

The patient was a 65-year-old woman presenting with fever, cough, and dyspnea due to COVID-19 pneumonia. She received antiviral agents, broad-spectrum antibiotics, and conservative treatment. Although her clinical condition improved, there was no significant improvement in portable chest X-ray results.

**Diagnoses:**

Due to concerns over the propagation of infection when transferring to patients for scanning and the need for excessive medical personnel to move patients, we moved a mobile chest computed tomography (CT) machine to an isolation ward for CT scanning.

**Interventions:**

We report our experience using mobile chest CT to effectively allocate medical resources and assess treatment response in patient with COVID-19 pneumonia.

**Outcomes:**

Follow-up mobile CT scans disclosed progressive resolution of the multifocal ground-glass opacities and mixed consolidations distributed peripheral to subpleural spaces. During the mobile chest CT scan, there were no adverse or unforeseen events. Three medical personnel were required to performed mobile chest CT, including a clinician, a nurse, and a radiologist.

**Lessons:**

As a result of using mobile chest CT on COVID-19 patients, the number of medical personnel required for CT scanning decreased by about 83%, rapid, and safe compared with a patient who performed conventional CT.

## 1 Introduction

In December 2019, an outbreak of severe acute respiratory syndrome coronavirus 2 (2019-nCoV) infections appeared in Wuhan, Hubei Province, China. To date, there is no specific treatment; therefore, coronavirus 2019 (COVID-19) poses a massive threat to global public health.^[1–4]^ Most of all, chest computed tomography (CT) is usually not necessary in patients with COVID-19; however, CT could play a role in diagnosing COVID-19 and monitoring treatment response.^[5]^ For patients with COVID-19, these advantages are counterbalanced by the need to transport patients outside a negative pressure isolation room and the increased expense and radiation doses associated with the CT procedure. Herein, we describe a case report with the novel use of mobile chest CT for assessing treatment response in patients with COVID-19 pneumonia as an example of effective utilization of medical resources.

## 2 Case presentation

On March 7, 2020, a 65-year-old woman who lived in Daegu, South Korea, presented to quarantine officials immediately, with a history of dyspnea, watery diarrhea, dry cough, fatigue, and low-grade subjective fever since March 3, 2020. Owing to the outbreak of COVID-19 in South Korea and the announcement of related symptoms, she was immediately placed in a quarantine ward and underwent examination. Although the patient reported no known contact with ill people during her stay in Daegu, specimens including oropharyngeal swab and sputum were collected for severe acute respiratory syndrome coronavirus 2 (SARS-CoV-2) real-time reverse-transcriptase-polymerase-chain-reaction (rRT-PCR) by the Korea Centers for Disease Control guidance.

She had a history of hypertension and rheumatoid arthritis with regular medical follow-up and took medicine. According to the records of quarantine officials, the vital sign of the patient showed body temperature of 38.3 °C and oxygen saturation (SpO_2_) of 88% to 90% under ambient air. Before seeking medical treatment in Daegu, she took over-the-counter medicine to release the symptoms. At that time, there was an absolute lack of a hospital in Daegu that could accommodate COVID-19 confirmed patient, so she was transferred to our institution on March 8, 2020. Her vital signs remained stable after admission to our hospital, aside from the exertional dyspnea, intermittent dry cough, and sore throat. The initial physical examination revealed a body temperature of 37.4 °C, blood pressure of 113/63 mm Hg, a pulse of 97 beats per minute, respiratory rate of 20 breaths per minute, and SpO_2_ of 93% while the patient was breathing supplement oxygen by nasal cannula at 3 L/min. Blood tests revealed lymphopenia (510 cells/μL [normal, 1500–3500 cells/μL]), monocytopenia (110 cells/μL [normal, 500–1000 cells/μL]), and elevated aspartate aminotransferase (95 IU/L [normal, 500–35 IU/L]), alanine aminotransferase (141 IU/L [normal, 5–40 IU/L]), C-reactive protein (23.5 mg/L [normal, 0–5 mg/L]), procalcitonin (0.25 ng/mL [normal, 0–0.1 ng/mL]), and lactate dehydrogenase (486 IU/L [normal, 135–225 IU/L]). The chest X-ray revealed bilateral perihilar infiltration and ill-defined patchy opacities at the right middle and lower lung fields, dominantly. On March 8, 2020, the oropharyngeal swab and sputum results were confirmed positive for SARS-CoV-2 by rRT-PCR assay.

Conservative treatments including antitussive agent, oxygen supplement, careful fluid infusion antiviral agent (lopinavir 200 mg plus ritonavir 50 mg, twice a day orally), and empiric antibiotics with ceftriaxone (2 g loading and 2 g, everyday intravenously) plus levofloxacin (750 mg, everyday intravenously) were administered from the first day of hospitalization.

**Figure 1:**
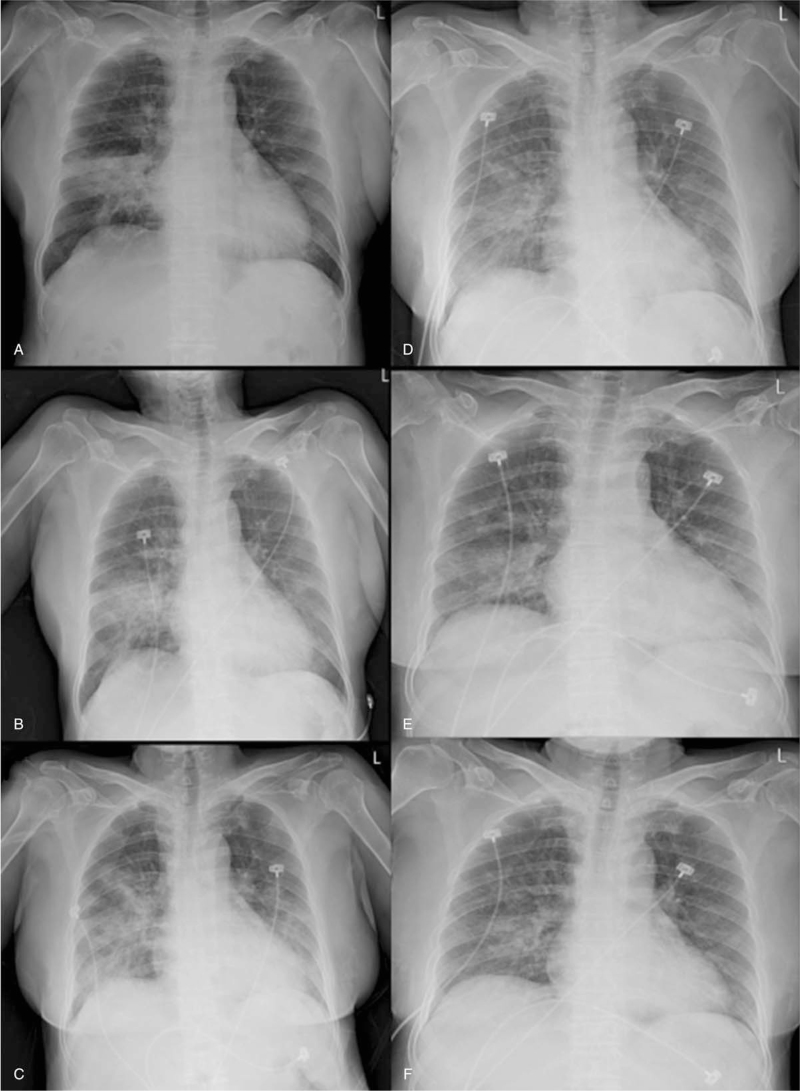
The results of serial chest X-rays in patients with COVID-19 pneumonia performed on illness day (A) 13 (hospital day 1), (B) 14, (C) 16, (D) 18, (E) 21, and (F) 23. COVID-19 = coronavirus 2019.

**Figure 2:**
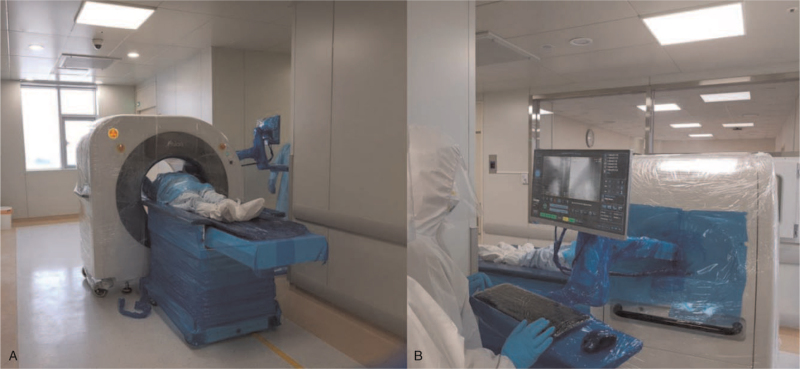
Mobile CT set up in the hallway in front of the isolation room. (A) Scene of COVID-19 patient mobile CT scan. (B) Scene of radiologic staff manipulates the mobile CT. COVID-19 = coronavirus 2019, CT = computed tomography.

**Figure 3:**
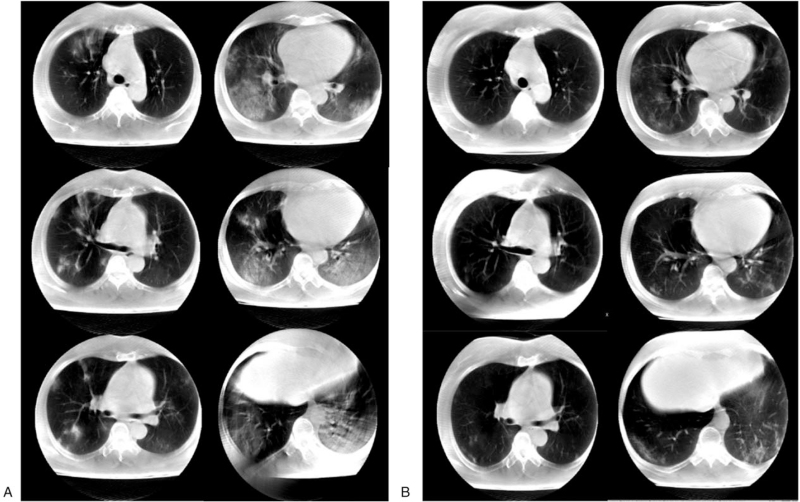
Mobile CT scan in patients with COVID-19 pneumonia. (A) The initial axial CT scans revealed multifocal ground-glass opacities and mixed consolidation distributed from peripheral to subpleural in both lungs. (B) Evidence of apparent resolution of the parenchymal lesions was identified in follow-up axial CT scans obtained on hospital day 9. COVID-19 = coronavirus 2019, CT = computed tomography.

**Figure 4:**
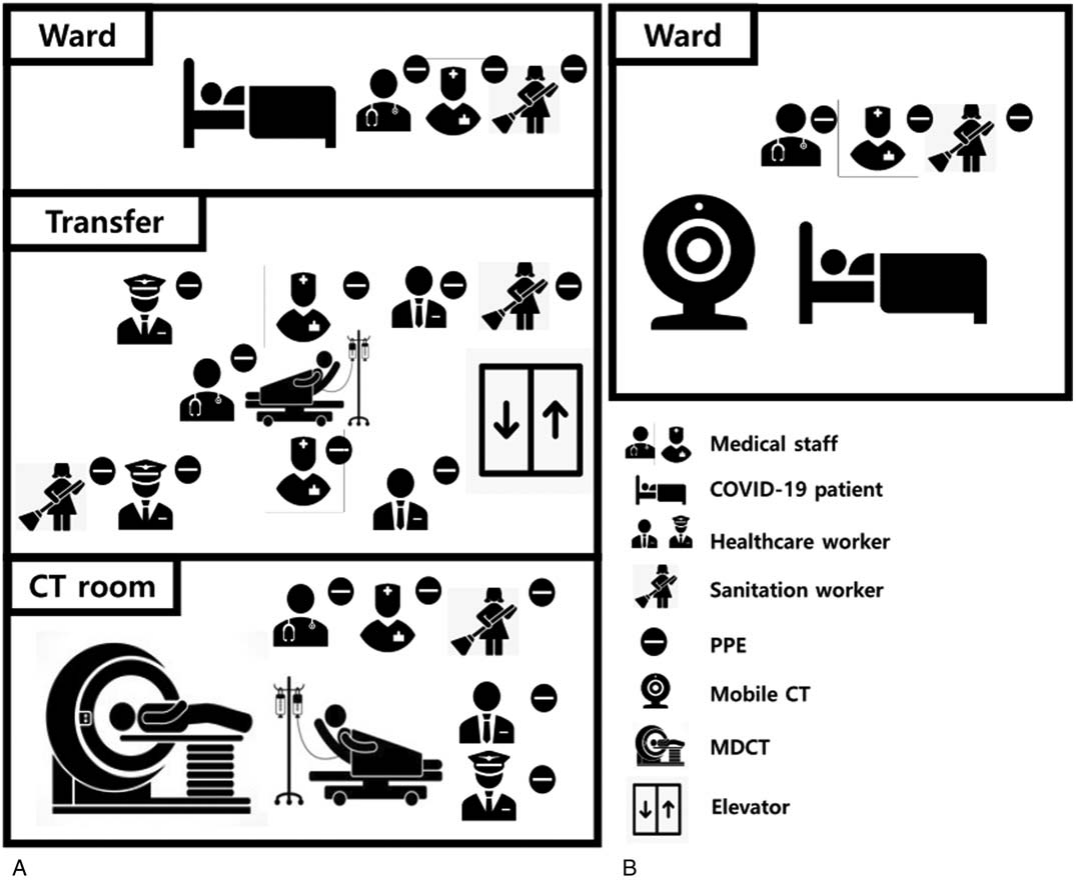
Illustration of medical personnel roughly required between conventional CT and mobile CT for CT scan (A) requirement workforce of conventional CT for CT scan (B) requirement workforce of mobile CT for CT scan. COVID-19 = coronavirus 2019, CT = computed tomography, MDCT = multi detector CT, PPE = personal protective equipment.

The results of serial chest X-rays performed on illness day 13 (hospital day 1), 14, 16, 18, 21, and 23 were inconsistent her symptom's improvement and revealed with no interval change of ground-glass opacity and slowing remission of worsening patches infiltrating at first hospital day (Fig. 1). Supplemental oxygen was discontinued on illness day 17, and her SpO_2_ improved to 95% to 98% under ambient air. Although her clinical condition improved, there was no significant improvement in chest X-ray results. She has intermittent exertional dyspnea, making it difficult to determine the transfer to the center of cohort isolation. To evaluate the patient's response to treatment, clinicians decided to take a CT scan. To address the enormous engagement of medical resources necessary to transport a patient with COVID-19 to receive a CT scan, our institution has implemented the use of a mobile CT for these patients (Fig. 2). For this purpose, we use a mobile volumetric cone-beam computed tomography (CBCT) (Phion2.0, NanoFocusRay Ltd., Iksan, Korea) for lung imaging.^[1]^ The mobile CT scans were performed on illness days 16 and 23. This machine uses an anode cone-beam X-ray source and a flat-panel detector with an amorphous silicon image sensor. The X-ray source can generate 50 to 130 kV and 4 to 18 mA with a 500 μm focal spot size.^[6]^ The field of view of the mobile CBCT for lung imaging is 236 mm (*XY*) × 176 mm (*Z*), and the voxel size is 230 μm^3^in 1024 (*XY*) × 1024 (*Z*). The shortest scan time of this volumetric CBCT is 7 seconds. The scanner weighs approximately 400 kg and is 1.66 m wide, 0.88 m deep, and 1.55 m tall. An iterative reconstruction algorithm is used for image reconstruction and for generating a preprocessed sinogram. The obtained images are preprocessed into 2-dimension (D) projection images in parallel to decrease image-processing time. The lung CT images are displayed using a 3D image viewer (Xelis, INFINITT, Korea). Initial axial CT scans disclosed multifocal ground-glass opacities and mixed consolidations distributed peripheral to subpleural spaces in both lungs, predominantly in both lower lobes. Follow-up axial CT scans revealed progressive resolution of the parenchymal lesions (Fig. 3). During the mobile chest CT scan, there were no adverse or unforeseen events. Three medical personnel were required to perform mobile chest CT, including a clinician, a nurse, and a radiologist. We also have experience with conventional CT on other patients at similar times for evaluation of treatment response. Although there was an unskilled portion and excessive protection, 17 medical personnel needed to move, scan, and secure the moving line (Fig. 4).

On illness day 20, her sputum turned undetectable for SARS-CoV-2 by rRT-PCR assay. However, the results of subsequent rRT-PCR of oropharyngeal swabs were repeatedly positive up to discharge day. The final culture results include blood, sputum, and urine, identified as negative on day 20. Also, all blood test results were normalized on illness day 26. Using her clinical conditions and CT results, she was then transferred to the center of cohort isolation on illness day 27 (March 29, 2020).

## 3 Discussion

In February 2020, the World Health Organization officially named the disease caused by this novel coronavirus as COVID-19.^[2]^ Coronaviruses belong to the *Coronaviridae* family, which infect both animals and humans.^[3]^ The disease spectrum of human coronaviruses varies from mild—similar to the common cold—to more severe diseases (such as Middle East respiratory syndrome and severe acute respiratory syndrome [SARS]).^[4]^ The virus is highly infectious and has spread rapidly.

The CT features reported in patients with COVID-19 pneumonia include bilateral and subpleural areas of ground-glass opacification, consolidation that affects the lower lung lobes, or both. In the intermediate phase of the infection, an irregular-paving pattern may be seen.^[5,7]^ Differences in the features of pneumonia caused by COVID-19 are actively studied.^[8–10]^ The CT has various advantages over conventional chest radiographs, including improved contrast resolution, tissue characterization capabilities, the ability to discriminate among multiple diseases, and monitoring of treatment response.

Health workers, including doctors, nurses, hospital administrators, sanitation workers, and others, are at the forefront of the outbreak response and are at high risk of contracting COVID-19. Risk factors include exposure to pathogens, long working hours, psychological pain, and fatigue.^[11,12]^ Various guidelines and protocols have been developed and include specific measures necessary to protect the safety and health of these professionals. When clinicians desire a CT scan of a patient with COVID-19 to monitor treatment response, they request assistance from the infection control department and seek guidance to determine the safest method for transporting patients with COVID-19. A time to carry out the CT scan is selected, typically at the end of the consultation hours, and the medical staff prepares to transport the patient for the CT scan. At least 1 medical staff and 1 healthcare worker enter the isolation area and assist in transporting the patient to a CT room. Transport includes moving to a removable bed, adjusting patient monitoring devices, and establishing a negative pressure tent. At least 4 healthcare workers are placed in line, and the secured patient is moved in a highly controlled environment. This transporting prevents bystanders or patients without COVID-19 from contacting the patient with COVID-19 being transported. Once in the CT room, at least 1 to 2 healthcare worker must seal the CT machine to prevent transmission of the 2019-nCOV virus from the patient. Also, at least 2 radiology staff member remains in the room for the CT scan. Sanitation workers clean and disinfect the isolation room, move transport lines, and clean the CT machine. All staff, healthcare workers, and cleaners must wear complete personal protective equipment to prevent disease transmission.

This mobile CT is small and has wheels to be moved, installed, and used anywhere with a wall outlet. The mobile CT is transferred to the patient's room to assist with evaluating the treatment response. Doing so reduces the time and cost associated with conducting a CT scan and reduces the risk of disease transmission to the public and healthcare workers. The mobile CT can also determine treatment response and determine pneumonia severity without leaving the negative pressure isolation room. It can be installed and used anywhere with shielding facilities and a wall outlet and does not require transformers. The mobile CT produces a radiation dose that is 20% of a conventional CT. The scanning of mobile CT may reduce radiation exposure to patients and healthcare professionals. The scanning time is approximately 7 seconds, and the reconstruction time takes around 40 seconds. We use mobile CT to monitor the treatment response in our patients with COVID-19 who receive conventional oxygen therapy. This process required only 2 medical personnel and 1 radiology staff member in an isolation room. Because of this, no disinfection was needed. In all, it takes approximately 20 minutes to confirm the image, including the time necessary to move the patient and conduct CT imaging.

CT is becoming increasingly crucial for the diagnosis and management of COVID-19 pneumonia. We expect the demand for CT scans to grow as the number of global cases of COVID-19 continues to increase. The value of CT would be further enhanced if it could define serial radiological changes or patterns that portend a poor outcome. Strict requirements for transporting, cleaning, and protective equipment necessary to scan patients with COVID-19 challenge healthcare systems. Because of this, CT scans are often reserved for critically ill patients or those with COVID-19 when there is uncertainty about diagnosis or treatment response. Emerging data will indicate if CT assists in disease monitoring and prognostication. The high efficiency and safe use of mobile CT will help healthcare workers treat patients with COVID-19 during this challenging time.

## Author contributions

**Conceptualization:** Jae Wan Jung, Chul Park.

**Data curation:** Jae Wan Jung, Hak Ryul Kim, Chul Park.

**Methodology:** Jae Wan Jung, Hak Ryul Kim, Kwon Ha Yoon, Chul Park.

**Resources:** Kwon Ha Yoon.

**Supervision:** Chul Park.

**Visualization:** Jae Wan Jung, Chul Park.

**Writing – original draft:** Jae Wan Jung.

**Writing – review & editing:** Jae Wan Jung, Hak Ryul Kim, Kwon Ha Yoon, Chul Park.
